# Department of Dermatology

**Published:** 1893-10

**Authors:** Isadore Dyer

**Affiliations:** Lecturer and Clinical Instructor Medical Department Tulane University, etc., New Orleans, La.


### Current Medical Literature.

### DEPARTMENT OF DERMATOLOGY.

[Dermatological Notes by Isadore Dyer, M. D., Lecturer and Clinical Instructor Medical Department Tulane University, etc., New Orleans, La.]

THYROID FEEDING IN SKIN DISEASES.—In the British *Journal of Dermatology* for September, Dr. Arthur T. Davies, of London, reports three cases of psoriasis and one of eczema, treated with tabloids of thyroid extract. Two of the cases of psoriasis were cured, one in eleven weeks after the disease began, and eight weeks after the thyroid treatment was commenced. The other case, of three years' standing, was cured after three months' treatment. The remaining case of psoriasis, and that of eczema, were much improved after treatment of one and three months, respectively.

The action of the extract, according to the author, is chiefly stimulant, causing at first increased desquamation, and a subsequent improvement in the tone of the skin. The contribution is interesting just now, when the testicular juice is being used in France with the same sort of result. Dr. Monnet cites (quoted in the *Jour. of Cut. & Gen. Urin. Dis.* for Sept.), three cases of ichthyosis, 14 instances of neuropathic eczema, etc., as happily modified by injections of the testicular fluid. In cases of cutaneous tropho-neuroses, Dr. M. believes injections of cerebrine preferable. (Communication of Dr. L. Brocq.)

GALLANOL is a purified form of gallol, and is offered as a substitute for chrysophanic acid and pyrogallic acid, both of which it resembles, chemically and therapeutically. For gallanol, it is claimed, that the disadvantages of these two acids are overcome, while the therapeutic properties are the same.

WARTS AND EXCRESCENCES.—A recent review of the methods of treating this class of growths gives the following:

Removal with the curette and cautery. Application of fuming nitric acid. Application of the tincture of thuja occidentalis. Exuberant vegetations should be dusted over with resorcin or salicylic acid. Salicylic acid plaster, 10 or 20 per cent., may be applied. For warty growths of the face this is suggested:

℞ Sublimed sulphur....................℥v

Pure concen..acetic acid............℥iiss

Glycerine...............  ..........℥ii

Mix and directions: Use with brush, or spread on with small pieces of linen, Apply at night and remove in morning.

Dr. D. Pacaud suggests solution of lemon juice for the relief of the pruritus of urticaria.

---

PROF. PITRES and Dr. Sabrazes (Archiv. clin. de Bordeaux, May, 1893), on examining three typical cases of syringomyelia, with classical cavities in the cord, found no evidence of the bacillus of Hansen. They conclude that syringomyelia and leprosy are two distinct diseases. (There is little question of the fact that there are types of syringomyelia of purely congenital origin; others of traumatic origin. It is not fair to exclude leprosy as a cause of this disease simply because the Hansen bacillus has not been isolated. Those cases of leprosy, most resembling syringomyelia, are found in communities where the disease is disappearing and the types are mild. The bacillus of Hansen is often missed in cases which clinically present all other signs of leprosy. It would be better, I think, to associate leprosy as one of the causes rather than condemn an alliance without further evidence.)

